# Impact of rear wheel axle position on upper limb kinematics and electromyography during manual wheelchair use

**DOI:** 10.1080/23335432.2018.1457983

**Published:** 2018-03-30

**Authors:** Hassanain Ali Lafta, Robert Guppy, Gemma Whatling, Cathy Holt

**Affiliations:** a Biomechanics Research Group, Health Technology and Digital World Theme, School of Engineering, Cardiff University, Wales, UK; b Biomedical Engineering Department, College of Engineering, Al-Nahrain University, Baghdad, Iraq

**Keywords:** Wheelchair propulsion, motion analysis, 3D kinematics, electromyography, rear wheel axle position

## Abstract

Manual wheelchair propulsion is an important form of mobility for people with lower limb disabilities. Changes in the wheelchair configuration can affect, range of motion (ROM) of the upper limb joints, muscle actions and system stability. The purpose of this study is to investigate the impact of adjusting wheelchair configurations on upper body joints kinematics and muscle recruitment for able-bodied non experienced manual wheelchair users through applying a marker-based 3D motion analysis technique. Ten healthy male subjects were characterised for three wheelchair configurations, set by adjusting the horizontal axle position of both rear wheels by (3 cm) and (6 cm) posteriorly from the original position set by the manufacturer. Selected 3D kinematic and surface electromyography (sEMG) parameters of the upper body joints and shoulder muscles were measured in the Cardiff University Motion Analysis Laboratory. During the propulsion trials, trunk flexion/extension, lateral bending and axial rotation were evaluated within the average range of (7.50°±1.4°), (5.91°±1.23°) and (7.01°±3.91°), respectively. Dominant shoulder abduction/adduction, flexion/extension and internal/external rotation were evaluated within the average range of (24.63°±6.38°), (17.31°±4.27°) and (40.02°±12.35°), respectively. Dominant elbow pronation/supination and flexion/extension were evaluated within the range of (15.49°±7.70°) and (34.37°±8.38°), respectively. Dominant wrist radial/ulnar deviation and flexion/ extension were evaluated within the average range of (29.82°±8.97°) and (53.59°±9.65°), respectively. With normalising the muscle EMG to the percentage of MVC activity, posterior deltoid had the highest average EMG muscle activity (11.43 ± 5.33) during the propulsion trials and at the three wheel adjustments relative to the other dominant shoulder muscles. Other average muscles activities were evaluated as (6.99 ± 2.37) for upper trapezius, (6.89 ± 2.51) for triceps brachii, (5.39 ± 2.95) for anterior deltoid, (3.26 ± 1.00) for biceps brachii and (3.14 ± 1.26) for pectoralis major as the lowest average activity. The findings of this study indicate that changing rear wheel axle position posteriorly is correlated with increasing the kinematic ROMs of the trunk and dominant upper limb and the sEMG activities of the muscles predominantly involved with the recovery phase of propulsion which could be linked with higher risks of musculoskeletal disorders. This knowledge may help professionals when designing and prescribing wheelchairs that are more proper to users’ functional characteristics, accordingly profiting them improved quality of life.

## Introduction

Manual wheelchair propulsion is an important form of mobility for people with lower limb disabilities to maintain their independence in activities of daily living and to become productive members of their communities (WHO, 2011). People who use a manual wheelchair depend upon their upper limbs for mobility during their activities of daily living. Upper limbs of wheelchair users are subject to unnatural loading conditions and repetitive use. As a result of greater than normal usage of the upper limbs, shoulder pain and pathology is common among manual wheelchair users (Boninger et al. 2002).

In an effort to gain a better understanding of the relationship between manual wheelchair propulsion and shoulder pain and injury, researchers and clinicians have conducted biomechanical analyses of wheelchair propulsion leading to identification of modifiable risk factors, which would hopefully aid in the development of prevention and treatment interventions (Odle 2014).

Wheelchair propulsion is basically described as two phases of hand and arm movement: the push phase and the recovery phase. During the push phase, the individual’s hands are in contact with the push rim of the wheelchair and there is special application of force to the rim to increase or maintain wheelchair velocity. The recovery phase occurs after the propulsive phase; the arms are brought back to a position where a new propulsive phase can begin (Sanderson and Sommer 1985). These definitions allow researchers to compare findings during the push phase with those of the recovery phase.

The use of a manual wheelchair suitable for a user’s individual characteristics and needs can improve their independence, sense of participation and quality of life. Many aspects relating to wheelchair configuration affect user actions in a manual wheelchair; determining the overall mobility performance. Changes in the wheelchair configuration can affect propulsion forces, the range of motion (ROM) of the upper limb joints and system stability. Ultimately, all these aspects determine how easy or difficult it is to propel a wheelchair in everyday mobility (Medola et al. 2014).

Several studies have shown the importance of seat/backrest assembly and the relative position of the rear wheels to the user in terms of the biomechanics of manual propulsion. Boninger et al. (2000) completed a study that showed axle position relative to the shoulder was associated with significant differences in push rim biomechanics. They found that with the axle further back relative to the shoulder there is more rapid loading of the push rim and increased stroke frequency was required. Boninger et al. (2000) suggested that providing users with a wheelchair with adjustable axle position and setting up the chair to meet the user’s needs could improve propulsion biomechanics and reduce the risk of secondary injuries as a result of wheelchair propulsion.

Mulroy et al. (2005) studied the effect of changing the fore-aft seat position on shoulder joint forces, moments and powers during three levels of effort of wheelchair propulsion. They found that the seat posterior position resulted in a statistically significant reduction in peak superior shoulder joint forces during free, fast and graded propulsion. They concluded that the posterior seat position may reduce the risk of rotator cuff tendinopathy (Mulroy et al. 2005).

Samuelsson et al. (2004) also studied the effect of rear wheel position on wheelchair propulsion and seating aspects. A more forward position of the rear wheel had a significant effect on stroke frequency and push angle. They also reported an increase in the weight distribution with the more forward position of the wheel. However, in their study they did not find any difference between the two wheel positions with respect to mechanical efficiency, estimated exertion, breathlessness, seating comfort, estimated propulsion qualities, pelvic position or activity performance (Samuelsson et al. 2004).

Freixes et al. (2010) also assessed the changes in speed, acceleration, stroke frequency and shoulder ROM in relation to four different axle positions. The study showed that the up and forward axle position resulted in an increase in speed and acceleration with a higher stroke frequency and a decreased shoulder ROM. The axle position of down and backward axle position resulted in a lower speed and acceleration with a lower stroke frequency and an increased shoulder ROM. Freixes et al. (2010) indicated that these were clinically important findings for wheelchair propulsion in their homes.

The improvement of manual wheelchair propulsion has become increasingly important as the population of individuals using wheelchairs is growing and requires efficient mobility to maintain the user’s independence and quality of life. This study aims to investigate the impact of adjusting wheelchair key configurations on upper limb joints kinematics and muscles recruitment during manual wheelchair propulsion. Three rear wheel axle positions were adjusted to elucidate the aspects of upper limb performance for able-bodied non-experienced manual wheelchair users during their daily mobility. Even though some researchers were worked on the manual wheelchair propulsion field, few studies were reported about a thorough simultaneous analysis of upper body three-dimensional kinematics and surface EMG analyses during propelling a manual wheelchair during mobility performance. Though there is similar work, but in the present study some potential aspects were applied, in terms of data collection procedures and experimental set-up, that provide a feasible robust acquisition of the manual wheelchair propulsion for able-bodied non-experienced users in spite of the time consuming and laborious measurement protocol.

Meanwhile, a major reason for preferring non-experienced user subjects was their feature of novice as they could determine their own self-selected speed and pattern of propulsion without any prior knowledge and experience influencing them. Experienced wheelchair users have already established their own motor behaviour, which may affect potential outcomes. Also, well-experienced subjects usually have their own customised wheelchair. The difference in wheelchair design leads to more complex factors as results are interpreted. Researchers use able-bodied subject pool to eliminate multiple interactions of variables in those with disabilities.

## Materials and methods

### Subjects

Ten healthy male able-bodied novice individuals, (eight right-arm dominant, mean age 38.00 ± 3.97 years, and body mass index 30.79 ± 4.41), with no previous experience with manual wheelchair propulsion and no history of shoulder pathology or instability, participated in this study after giving their informed consents.

### Experimental protocol

Subjects were asked to propel a manual self-propelled wheelchair along a 10 metre linear path in the centre of the motion capture area across the Motion Analysis Laboratory at Cardiff University, while the motions of their trunk and dominant upper limb were measured using a motion analysis system (Oqus Cameras and QTM Software, Qualisys, Sweden). For five trials per subject, five consecutive strokes occurring during steady-state propulsion were included in the analysis. The start-up and stopping pushes were excluded. Before the propulsion measurements, subjects were given 5–10 min to get familiar with using the hand-rim wheelchair with their steady self-selected speed and propulsion pattern.

Controlling the stroke patterns for non-experienced manual wheelchair users will require many training pre-sessions. Richter et al. (2007) studied the stroke patterns of 25 individuals with paraplegia propelling their own wheelchairs at self-selected speeds on a treadmill. They didn’t find significant differences in the push rim biomechanics between the different stroke patterns based on propulsion mechanism.

The relationship between push rim biomechanics and relative rear wheel axle position was investigated using the kinematic analysis of the trunk and dominant upper limb joints and sEMG recording of dominant shoulder muscle activation while adjusting the horizontal axle position of both right and left rear wheels by (3 cm) and (6 cm) displacements posteriorly from the manufacturer’s position while keeping the seat height constant according to the manufacturer’s specification, i.e. (48 cm) (Invacare 2011).

**Figure 1.**
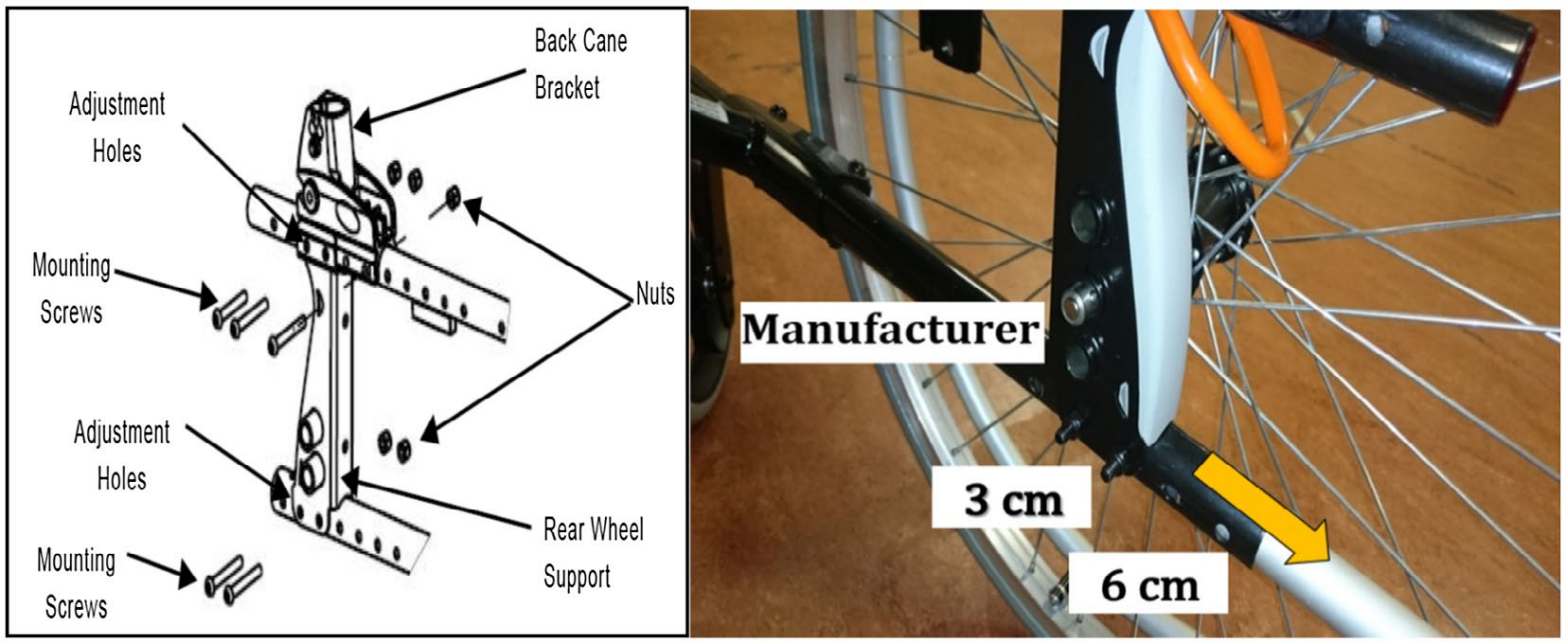
Schematic diagram about the manufacturer and posterior displacements of the rear wheel axle position, (Invacare 2009)

These displacements were made according to the set up instructions mentioned by the product adjustments manual. The existing adjustment holes on the used wheelchair frame were incremented from the manufacturer’s wheel hub by (1 cm) between each other (Invacare 2009), see Figure 1. Therefore, we selected the three positions, (the manufacturer’s set, (3 cm) and (6 cm) displacements), in order to achieve consistent measurements. Providing wheelchair users with adjustable axle position and then fitting the user to the wheelchair can improve propulsion biomechanics and likely reduce the risk of injury (Boninger et al. 2000).

### Marker placement

**Figure 2.**
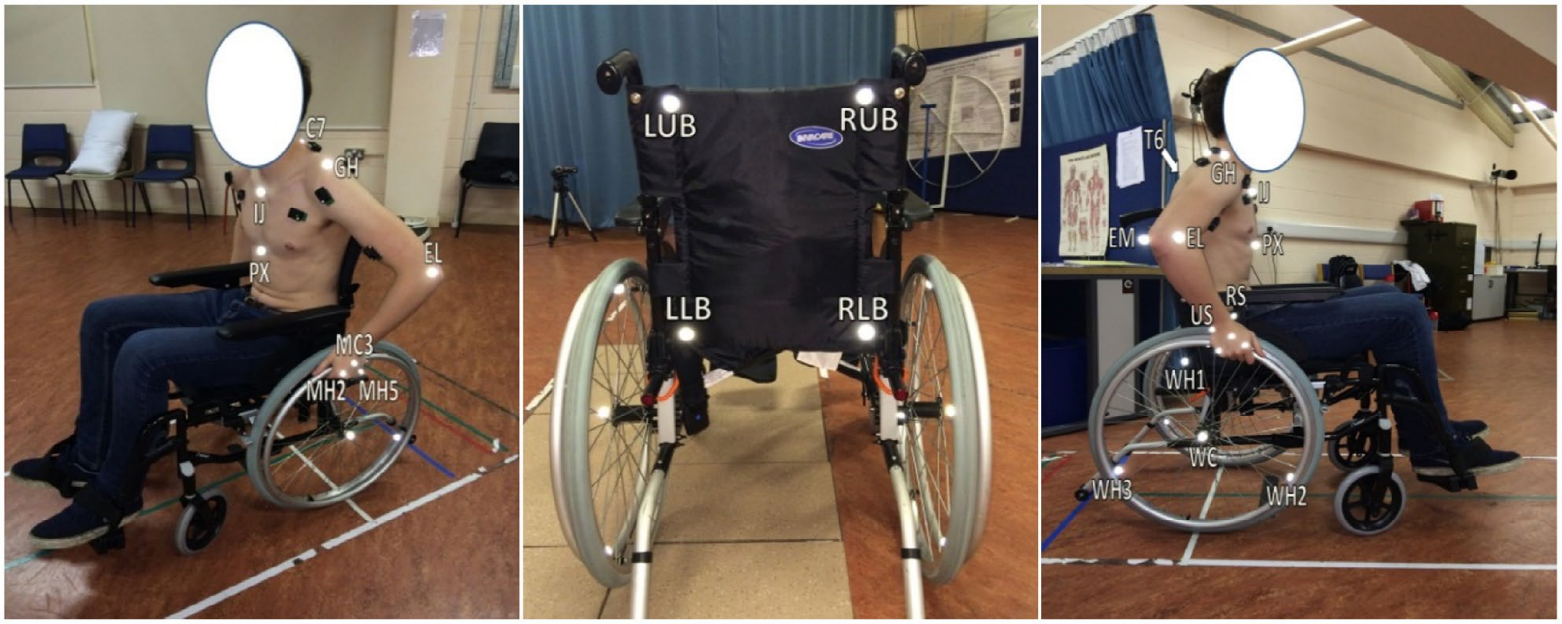
Markers set placement on the trunk, right upper limb and the wheelchair

A set of twenty retro-reflective markers were attached to the skin overlying specific bony landmarks of the thorax, right and left upper arms, forearms and hands. These bony landmarks are recommended by the International Society of Biomechanics (ISB) (Wu et al. 2005), to establish body segment and joint coordinate systems. An additional twelve markers were placed on the wheelchair’s back rest and on both right and left wheels to identify a local reference system. The bony landmarks were identified by means of palpation. Markers were attached onto the landmark using double-sided tape. During the bony landmarks identification process, subjects were asked to adopt the neutral position that is sitting down straight on the wheelchair, maintain both arms by the side of the body with elbows flexed at 90° and both hands pronated. The trunk markers included the suprasternal notch (IJ), xiphoid process (PX), spinal processes of C7 and T6 vertebrae. The upper arm markers included the acromion (GH), humeral medial (EM) and lateral (EL) epicondyles. The forearm markers included the humeral epicondyles and the radial (RS) and ulnar (US) styloids. The hand markers include the 2nd (MH2), 3rd (MC3) and 5th (MH5) metacarpals. The glenohumeral joint centre was calculated from the marker on the acromion by regression method adopted by Campbell et al. 2009; which was used because scapular motion tracking was not considered in this analysis. An additional four markers were placed on the wheelchair’s back rest corners and eight on both right and left wheels (one on each wheel’s hub and three markers placed around the inside edge of each wheel rim with 120° interval around the wheel. i.e. at 0°, 120° and 240° positions, respectively) to identify a local reference system, see Figure 2.

### Six degrees of freedom analysis

**Figure 3.**
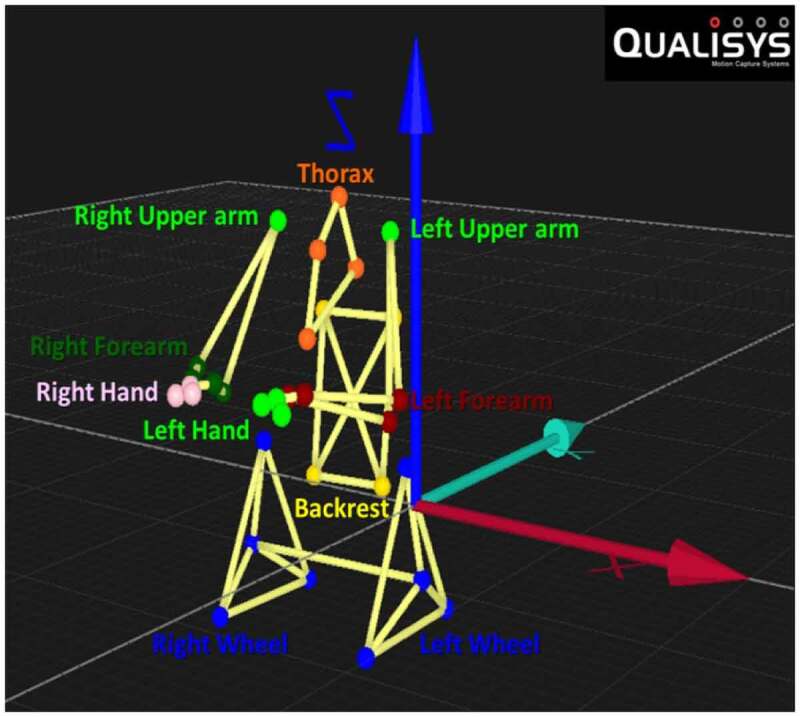
A QTM view of six degrees of freedom model of the trunk, right and left upper limbs and the wheelchair (backrest and wheels) during neutral position

In terms of establishing six degrees of freedom (6 DOF) analysis for three-dimensional kinematics, each upper limb is modelled as a four segments linked system that consists of trunk, upper arm, forearm and hand. Each segment is considered as a single rigid body. 6 DOF data (roll, pitch, yaw, x, y, z) were calculated according to the ISB recommendations. Segment coordinate systems were determined for trunk and right and left upper limbs segments (QTM Software, Qualisys, Sweden) see Figure 3. Trunk movement was determined with respect to the laboratory’s global coordinate system (GCS), upper arm movement with respect to the trunk, forearm movement with respect to the upper arm, and hand movement with respect to the forearm using Euler angle notation and a sequence of ZXY rotations of the trunk, upper arm and hand, and ZYX rotations of the forearm. Although it was recognised that the shoulder complex motion involves the intricate linkages between the humerus, scapula and thorax, kinematic distinction between these rigid bodies were not considered per this study, and instead, only the gross motion of the humerus (upper arm) relative to the thorax (trunk) was considered in terms of humero-thoracic rotation. The rotation order of the humerus relative to the thorax was ZXY order, rather than the YXY order. As, in the latter order, gimbal lock occurs when the elevation of the upper arm will tend to zero degree. Also, both the axial rotation and the plane of elevation change greatly giving rise to extreme values (Doorenbosch et al. 2003). Flexion, adduction and internal rotation of the shoulder joint were defined as positive values.

Following the ISB recommendations, the axes of each segment’s coordinate system are aligned such that the X-axis directs anteriorly, the Y-axis directs superiorly and the Z-axis directs laterally towards the right side. A local coordinate system was determined for each segment. To define an orthogonal local coordinate system to a rigid body segment, the coordinates of three non-collinear points within the segment must be known. The coordinate system is thus defined by the three mutually orthogonal unit vectors. The joint angles were determined by the relative motion between two adjacent segments, distal relative to proximal, following the right hand rule with the X-axis as the abduction/adduction axis, the Y-axis as the internal/external rotation axis and the Z-axis as the flexion/extension axis (Zatsiorsky 1998).

### Muscle activation analysis

In terms of analysing the sEMG activity of shoulder stabilising muscles during manual wheelchair propulsion, sEMG signals were recorded on six recruited shoulder muscles of each healthy volunteer’s dominant arm while performing their manual wheelchair propulsion.

Subjects were prepared for the placement of sEMG electrodes by shaving the skin of each electrode site, cleaning it carefully with an alcohol wipe and lightly abrading it. DELSYS Trigno wireless sEMG system was used to measure the sEMG signals of these muscles, which were recruited for their well-known contribution to wheelchair propulsion. The six muscles were: anterior deltoid, middle deltoid, posterior deltoid, sternal head of the pectoralis major, biceps brachii, triceps brachii and the upper part of the trapezius (Louis and Gorce 2010).

**Figure 4.**
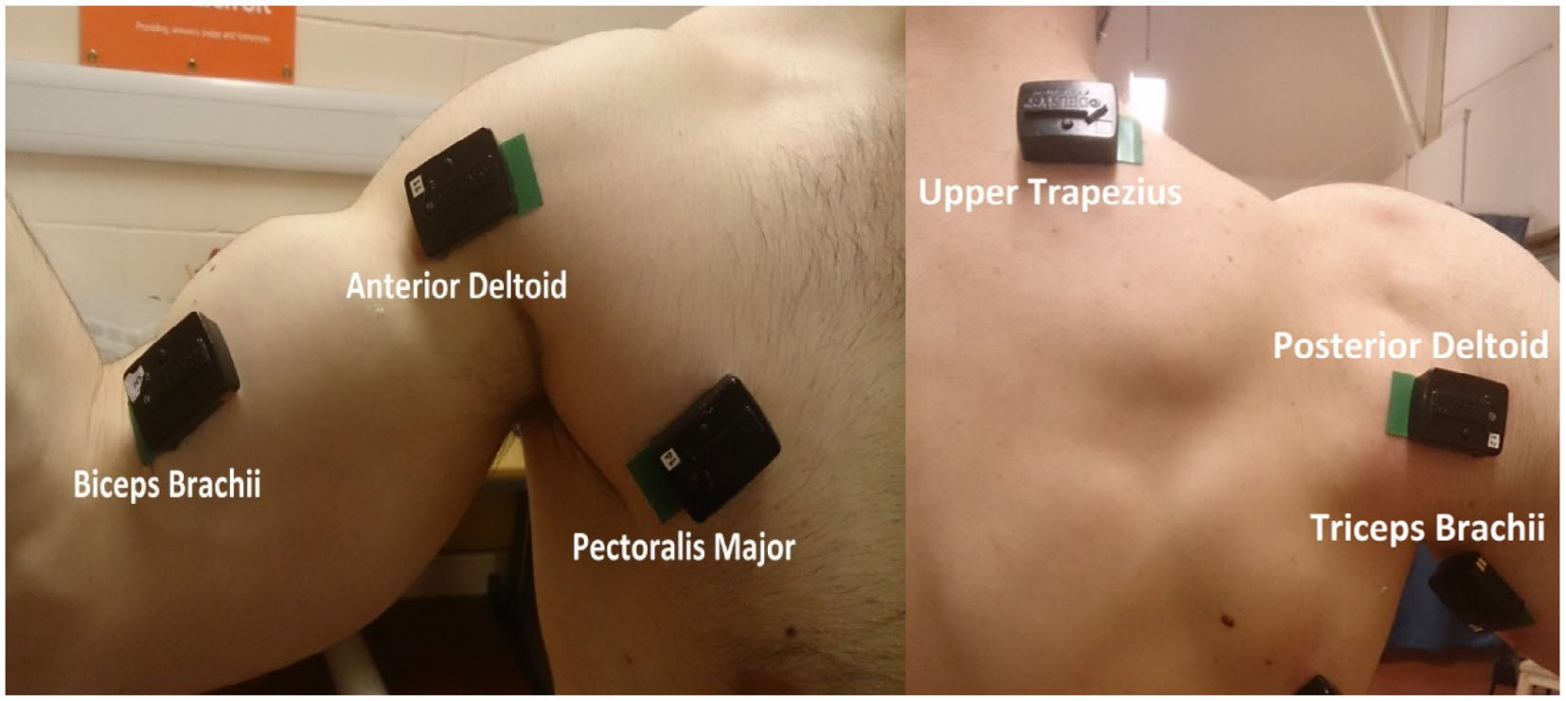
DELSYS Trigno wireless surface EMG electrodes placed over six dominant shoulder muscles

sEMG electrodes were positioned along the midline of the muscle belly in the direction of the muscle fibres because this reduces the likeliness of the electrode detecting crosstalk from adjacent muscle fibres. Electrode placement was confirmed by testing elevation (anterior and posterior deltoid), external rotation (upper trapezius and posterior deltoid), internal rotation (pectoralis major) and arm flexion (biceps brachii and triceps brachii). The electrode placement locations were selected based on (Perotto 2011), see Figure 4.

### EMG normalisation

**Figure 5.**
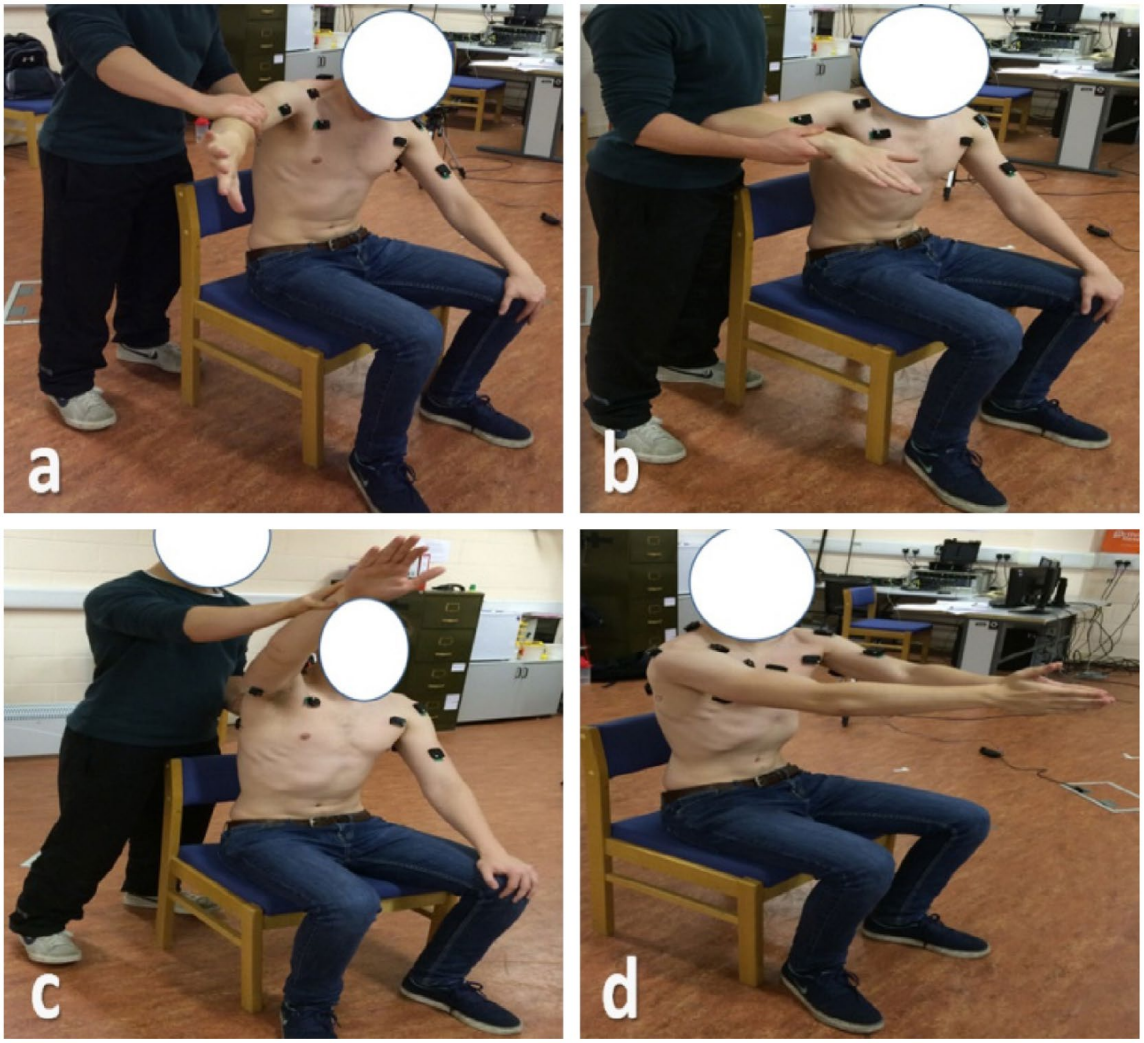
Shoulder muscles normalisation standard tests. (a) Empty can, (b) Internal rotation 90°, (c) Flexion 125° and (d) Palm press

Prior to the collection of propulsion data, a set of tasks were performed by each subject for inducing a maximum voluntary contraction (MVC) in each muscle for normalising the sEMG activity of the shoulder muscles reliably. These tasks were suggested by Boettcher et al. (2008) to maximally and reliably activate all shoulder muscles tested. Therefore, this study suggests that these tests be adopted as standard tests for generating a maximum voluntary contraction MVC for normalisation in future sEMG research at the shoulder. These four tests are illustrated as follows: (i) the *empty can* test position with the shoulder abducted to 90° in the plane of the scapula, internal humeral rotation, and elbow extended. The arm is abducted as resistance is applied at the wrist, see Figure 5(a); (ii) the *internal rotation 90°* test position with the shoulder abducted to 90° in the plane of the scapula, neutral humeral rotation, and elbow flexed 90°. The arm is internally rotated as resistance is applied at the wrist, see Figure 5(b); (iii) the *flexion 125°* test position with the shoulder flexed to 125° as resistance is applied proximal to the elbow and at the inferior angle of the scapula, attempting to de‐rotate the scapula with the subject sitting in an erect posture with no back support, see Figure 5(c); (iv) the *palm press* test position with the shoulders flexed to 90° bilaterally, the heel of the hands together, elbows flexed 20°, and then the arms horizontally adducted, see Figure 5(d). The collected sEMG data are normalised to the MVC taken on the same day of those particular trials. This was to make the normalised data more reliable because the MVC was recorded with exactly the same electrode configuration, positions and conditions as was used in the propulsion trials (Boettcher et al. 2008).

Using maximum voluntary contraction is a highly reliable method to normalise EMG data and can be used to compare activity between muscles, between tasks and between individuals. The four MVC tasks were performed together, separated by at least 30 s between each one. Based on the repeatability between test measures, two repetitions of the tests were performed separated, by at least 3 min to reduce any possible fatigue effects. The maximum values obtained from the processed signals during all repetitions of the test were used as the reference value for normalising the EMG data, processed in a similar manner from the muscles of interest. Boettcher et al. (2008) have shown that multiple tests can produce maximum recording from any given muscle and that no specific test produces maximum recording from a given muscle in all individuals tested.

### Data processing

The kinematic data for each marker trajectory were collected at a sampling rate of (60 Hz), identified, processed and exported, (as per Figure 3), for analysis from QTM. For each subject, wheelchair stroke cycles were analysed to compute the mean group parameters of interest. Peak joint angles (maximum and minimum) were identified from each cycle and used to compute the ranges of motion (ROMs).

The sEMG signals were recorded at a sampling frequency of (1080 Hz). Each raw sEMG data-set was exported to MATLAB (Mathworks, Massachusetts, United States, 2016) for signal analysis and post-acquisition processing. Raw EMG signals from the propulsion trials and MVC tasks were pre-amplified, high-pass filtered by a Butterworth fourth order filter at (20 Hz), full wave rectified, and low pass filtered with a fourth order Butterworth filter at (500 Hz). Muscle activation was described as the linear envelope of the signal. This type of treatment eliminates ambient noise through the high-pass filter, and smoothens the curve through full-wave rectification and the low pass filter, thus creating the linear envelope, (Boettcher et al. 2008).The high pass filter was used to remove the low frequency signals associated with soft tissue artefacts caused by skin movement due to muscle contraction. The low pass filter was used to remove the high frequency signals associated with interruption caused by the electrodes being subject to a force that may be applied by the observer’s resistance at the participant’s scapula, elbow and wrist during performing the MVC tasks. Both the high and low pass filters were utilised in a band pass filter. Once the signal processing had been applied, the processed sEMG data was normalised to the MVC tasks performed so as to be presented as a percentage of the maximum contraction that was established in the MVC four tasks against time.

## Results

All the selected kinematic and sEMG data were averaged from five propulsion trials performed by each volunteer then averaged again for the ten volunteers for each wheelchair configuration. The mean and standard deviation values of the trunk and dominant upper limb joint kinematics, as well as the dominant shoulder muscle normalised activities were analysed. To compare the results between the different wheelchair configurations, parametric repeated measures were used since the data were normally distributed. The statistical analyses were employed by using Microsoft Excel two ways ANOVA. P-value was calculated. Descriptive statistics were calculated for each variable. The level of significance was set at *p* < 0.05 for all statistical analyses, see Tables 1 and 2.

**Table 1.** Kinematic ROM angles of trunk and dominant upper limb joints with the manufacturer’s rear wheel axle position and (3 cm) and (6 cm) backward displacements. All the joint ROM angles are presented as group mean and standard deviation values in degrees

Dominant Upper Limb Rigid Segment Kinematics		Joint ROM Angles (°) at Rear Wheel Axle Position			*P* value *<0.05
		Manufacturer’s position	3 cm Backward Displacement	6 cm Backward Displacement	
Trunk	Flexion / Extension	7.504 ± 1.397	8.018 ± 2.563	8.057 ± 2.685	0.788
	Lateral Bending	5.905 ± 1.232	6.136 ± 1.235	6.345 ± 1.647	0.734
	Axial Rotation	7.012 ± 3.909	7.143 ± 3.452	7.279 ± 2.571	0.974
Dominant Shoulder	Adduction / Abduction	24.627 ± 6.383	25.897 ± 7.472	28.228 ± 5.724	0.01*
	Internal / External Rotation	17.314 ± 4.274	18.724 ± 4.759	20.533 ± 7.081	0.004*
	Flexion / Extension	40.021 ± 12.348	43.029 ± 7.096	46.945 ± 10.606	0.001*
Dominant Elbow	Pronation / Supination	15.493 ± 7.703	18.876 ± 6.828	19.521 ± 6.187	0.416
	Flexion / Extension	34.366 ± 8.376	35.977 ± 11.185	40.137 ± 10.297	0.44
Dominant Wrist	Radial / Ulnar Deviation	29.816 ± 8.792	35.535 ± 8.229	36.884 ± 8.162	0.139
	Flexion / Extension	53.591 ± 9.652	55.877 ± 13.328	57.036 ± 16.066	0.757

^*^ Significantly different to both other rear wheel axle positions (*p* < 0.05).

**Table 2.** Muscle activation in terms of normalised EMG percentage MVC of dominant shoulder muscles with the manufacturer’s rear wheel axle position and (3 cm) and (6 cm) backward displacements. All the muscles normalised EMG %MVC are presented as group mean and standard deviation values

Dominant Shoulder Muscle	Normalised EMG %MVC at Rear Wheel Axle Positions			*P* value *<0.05
	Manufacturer’s position	3 cm Backward Displacement	6 cm Backward Displacement	
Anterior Deltoid	5.392 ± 2.954	4.495 ± 2.947	4.258 ± 2.41	0.417
Posterior Deltoid	11.425 ± 5.33	11.846 ± 6.738	12.298 ± 6.074	0.861
Biceps Brachii	3.263 ± 1.001	4.116 ± 1.39	4.305 ± 1.63	0.197
Pectoralis Major	3.143 ± 1.259	3.456 ± 1.426	3.57 ± 1.531	0.762
Upper Trapezius	6.985 ± 2.366	7.242 ± 4.115	8.424 ± 6.24	0.622
Triceps Brachii	6.886 ± 2.514	6.189 ± 3.272	5.203 ± 2.793	0.190

Notes:

^*^ Significantly different to both other rear wheel axle positions (*p* < 0.05).

Moreover, all the data were tested for normality through using the Shapiro–Wilk expanded tests with using the Q–Q plots for the normal distribution comparison. When *P*-value was more than the chosen level of significance (i.e. 0.05), then an evidence that the data tested were from a normally distributed population. While when the P-value was less than 0.05 then the null hypothesis that the data are normally distributed is rejected.

However, No significant differences were identified between the trunk and dominant upper limb kinematics and dominant shoulder muscle sEMG activities, significant differences were indicated in the dominant shoulder average ROMs for abduction/adduction (*p* = 0.01 < 0.05), internal/external rotation (*p* = 0.004 < 0.05) and flexion/extension (*p* = 0.001 < 0.05) average kinematic ROM produced by the subjects during the three rear wheel axle adjustments, see Table 1.

**Figure 6.**
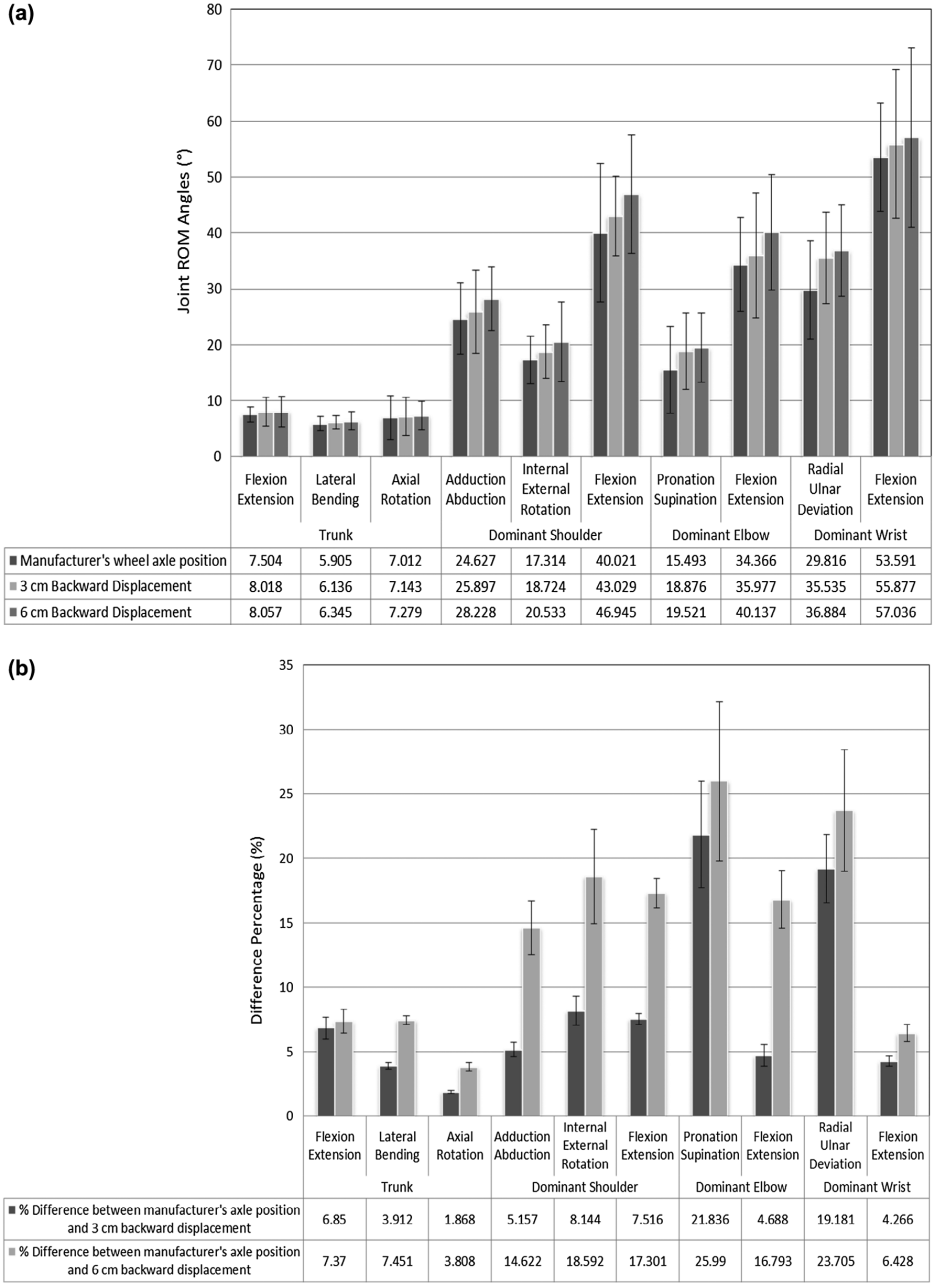
(A) Kinematic ROM angles of trunk and dominant upper limb joints with three wheel axle positions. All the joint ROM angles are presented as group average values. (B) Difference percentage of kinematic ROM angles of trunk and dominant upper limb joints between the manufacturer’s wheel axle position and (3 cm) and (6 cm) backward displacements

Three-dimensional trunk and dominant upper limb joints kinematics during manual wheelchair propulsion has been evaluated, see Figure 6.

It was found that the average and standard deviation values to be within the range of (7.50° ± 1.4°) for trunk flexion/extension, (5.91° ± 1.23°)for trunk lateral bending and (7.01° ± 3.91°) for trunk axial rotation. Gagnon et al. (2015) reported trunk flexion/extension motion to be within the range of (5.88° ± 2.12°).

Dominant shoulder abduction/adduction, flexion/extension and internal/external rotation were evaluated within the range of (24.63° ± 6.38°), (17.31° ± 4.27°) and (40.02° ± 12.35°), respectively. Boninger et al. (1998) reported shoulder motion at speed of (1.3 m/sec) to be within the range of (75°) for flexion/extension, (26°) for abduction/adduction and (37°) for internal/external rotation. While Soltau et al. (2015) reported shoulder motion to be within the range of (72.6°) for flexion/extension, (67.9°) for abduction/adduction and (22.8°) for internal/external rotation.

Dominant elbow pronation/supination and flexion/extension were evaluated within the range of (15.49° ± 7.70°) and (34.37° ± 8.38°), respectively. Soltau et al. (2015) reported elbow motion to be within the range of (45.7°) for flexion/extension and (28.8°) for pronation/supination (forearm rotation).

Furthermore, dominant wrist radial/ulnar deviation and flexion/extension were evaluated within the range of (29.82° ± 8.97°) and (53.59° ± 9.65°), respectively. Boninger et al. (2004) reported wrist motion at low speed (0.9 m/sec) to be within the range of (50.3°) for flexion/extension and (44.6°) for radial/ulnar deviation. Also, Crespo-Ruis et al. (2011) reported wrist motion in their study about wheelchair basketball to be within the range of (27.18°) for flexion/extension and (21.46°) for radial/ulnar deviation.

While displacing the axle position backward (posteriorly), the distance between the user’s centre of gravity and the rear wheel axle is increased making the wheelchair harder to push. Therefore, an increased kinematic ROM is required for the users to reach their starting position with the push rim to perform the propulsion.

Mulroy et al. (1996) identified two synergies of shoulder muscle function during wheelchair propulsion. The push phase synergy was dominated by muscles with shoulder flexion (anterior deltoid, pectoralis major), external rotation (supraspinatus, infraspinatus) and scapular protraction (serratus anterior) functions. While, the recovery phase synergy was dominant extension (posterior deltoid), abduction (medial deltoid, supraspinatus), internal rotation (subscapularis) and scapular retraction (middle trapezius) (Mulroy et al. 1996).

**Figure 7.**
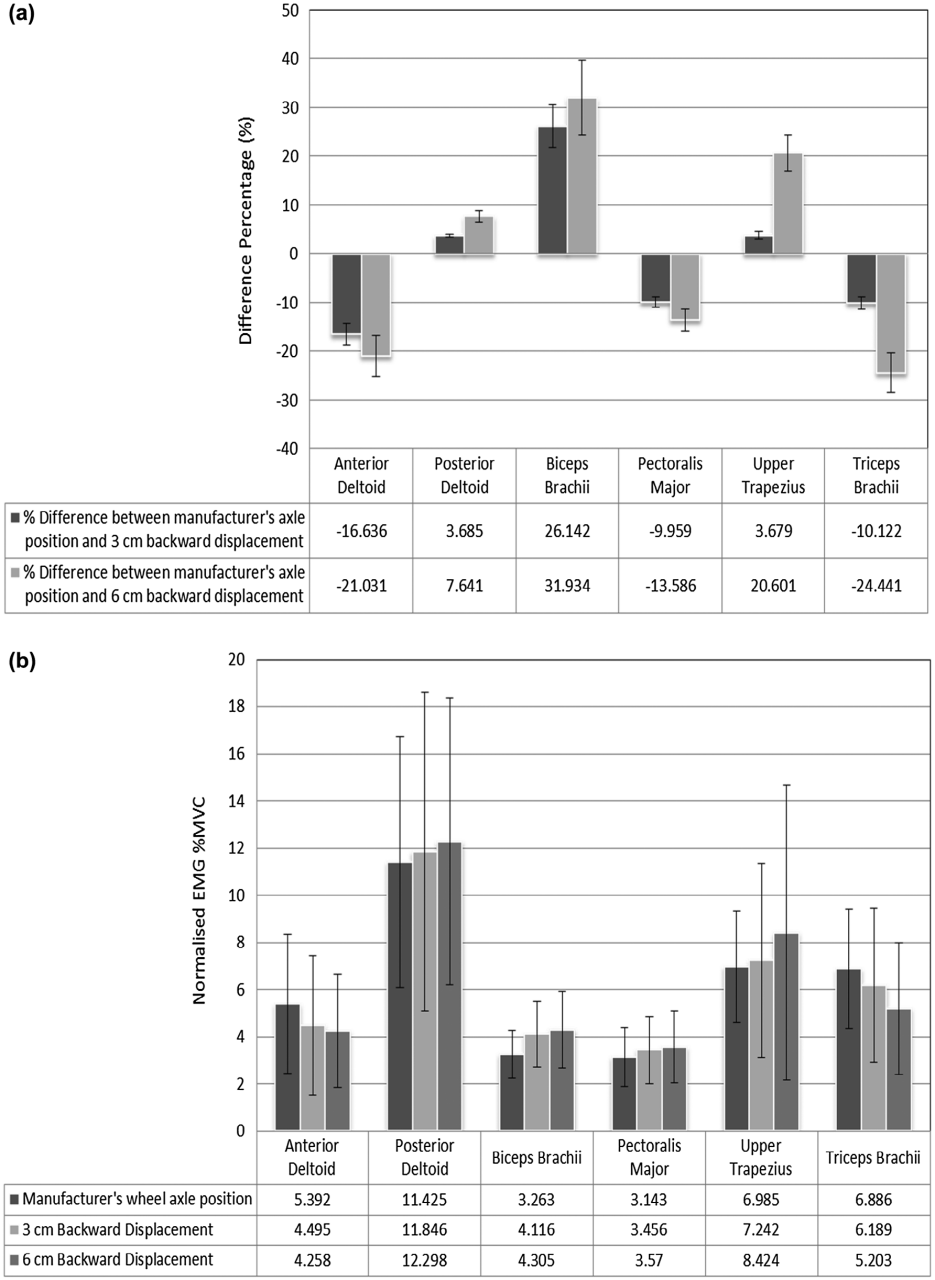
(A) Muscle activation in terms of normalised EMG percentage MVC of dominant shoulder muscles with three wheel axle positions. All the muscles normalised EMG %MVC are presented as group average values. (B) Difference percentage of muscle activation in terms of normalised EMG percentage MVC of dominant shoulder muscles between the manufacturer’s wheel axle position and (3 cm) and (6 cm) backward displacements

In this study, we tested pectoralis major, anterior deltoid muscles in the push phase synergy muscle group and posterior deltoid and upper trapezius in the recovery phase synergy muscle group. The EMG activities evaluated in this study were lower than the activities reported by other studies (Mulroy et al. 1996) due to the differences of participant populations between the studies. With normalising the muscle EMG to the percentage of MVC activity, our results showed that the posterior deltoid had the highest average EMG muscle activity (11.43 ± 5.33) during the propulsion trials and at the three wheel adjustments relative to the other dominant shoulder muscles. The other average muscle activities were evaluated as (6.99 ± 2.37) for upper trapezius, (6.89 ± 2.51) for triceps brachii, (5.39 ± 2.95) for anterior deltoid, (3.26 ± 1.00) for biceps brachii and (3.14 ± 1.26) for the pectoralis major as the lowest average activity, see Figure 7.

It was concluded by Schantz et al. (1999) that the biceps brachii and triceps brachii, anterior deltoid and pectoralis major muscles could be anticipated to propel the wheelchair forward, whereas the posterior deltoid and trapezius muscles could be expected to play a role, especially during the recovery phase. However, individual differences exist.

This high activation on average for the muscles predominantly involved with the recovery phase could be associated with the increase in the shoulder ROM required for the volunteer to reach their starting position on the hand rim. Also, the decreased activation on average for the muscles predominantly involved with the push phase could be associated with the increased upper limbs kinematics and the muscles pre-stretch potentiation.

Moreover, the findings of this study indicate that the general order of muscle activation is of, first, the biceps brachii, thereafter the pectoralis major and anterior deltoid, and then the triceps brachii muscles during the push phase of propulsion, and this is in agreement with other studies (Mulroy et al. 1996).

The impact of rear wheel axle position on the kinematic and EMG outcomes was quantified in terms of the difference per cent that indicates the change (increase or decrease) of these outputs as the rear axle moved posteriorly from the manufacturer’s position to 3 cm and 6 cm displacements separately. This was formulated as follows:

$$\%\text{Difference}=\left\{\frac{\text{Outcome during backward displacement}}{\text{Outcome during manufacturer}’\text{s axle position}-1}\right\}*\%100$$

The findings of this study indicate that the backward displacing rear wheel axle position led to be a significant factor affecting the trunk and dominant upper limb kinematic behaviour. For the trunk, the flexion/extension ROM increased by (6.85 ± 0.84%) and (7.37 ± 0.92%), lateral bending ROM increased by (3.92 ± 0.25%) and (7.45 ± 0.34%), axial rotation ROM increased by (1.87 ± 0.12%) and (3.81 ± 0.34%) as the horizontal axle displacement was moved from the manufacturer’s position to (3 cm) and (6 cm), respectively. For the dominant shoulder; the abduction/adduction ROM increased by (5.16 ± 0.57%) and (14.62 ± 2.10%), internal/external rotation ROM increased by (8.14 ± 1.11%) and (18.59 ± 3.66%), flexion/extension ROM increased by (7.52 ± 0.43%) and (17.3 ± 1.14%) as the horizontal axle displacement was moved from the manufacturer’s position to (3 cm) and (6 cm), respectively. In a similar manner, for the dominant elbow; the pronation/supination ROM increased by (21.84 ± 4.11%) and (25.99 ± 6.2%), flexion/extension ROM increased by (4.69 ± 0.84%) and (16.79 ± 2.23%) as the horizontal axle displacement was moved from the manufacturer’s position to (3 cm) and (6 cm), respectively. For the dominant wrist, the radial/ulnar deviation ROM increased by (19.81 ± 2.64%) and (23.71 ± 4.72%), the flexion/extension ROM increased by (4.27 ± 0.38%) and (6.428 ± 0.67%), as the horizontal axle displacement was moved from the manufacturer’s position to (3 cm) and (6 cm), respectively, see Figure 6.

Further, this study indicates that the backward displacing rear wheel axle position led to increase the average %MVC magnitudes of the muscle activation associated with the prime movers in the recovery phase of propulsion. The average %MVC magnitude of the posterior deltoid activity increased by (3.69 ± 0.26) and (7.64 ± 1.14) and for the upper trapezius increased by (3.68 ± 0.74) and (20.60 ± 3.64) as the horizontal axle displacement was moved from the manufacturer’s position to (3 cm) and (6 cm), respectively. In contrast to this, the average %MVC magnitudes of the muscles associated with the push phase was decreased. The average %MVC magnitude of the anterior deltoid decreased by (16.64 ± 2.23) and (21.03 ± 4.18) and for the pectoralis major decreased by (9.96 ± 1.13) and (13.59 ± 2.22) as the horizontal axle displacement was moved from the manufacturer’s position to (3 cm) and (6 cm), respectively. While the average %MVC magnitude of the biceps brachii activity was increased by (26.14 ± 4.39) and (31.93 ± 7.63) and for the triceps brachii decreased by (10.12 ± 1.30) and (24.44 ± 4.11) as the horizontal axle displacement was moved from the manufacturer’s position to (3 cm) and (6 cm), respectively, see Figure 7.

## Discussion

Manual wheelchair users depend on their upper limbs for mobility during their activities of daily living. However, handrim wheelchair propulsion is a physically straining form of ambulation as a consequence of a high load on the shoulder complex. A population of ten healthy subjects was characterised for three wheelchair configurations, set by adjusting the horizontal axle position of both rear wheels by (3 cm) and (6 cm) posteriorly from the original position that was set by the manufacturer (Invacare 2011).

A protocol for 3D kinematic measurements of the upper limb joints was applied using a six degrees of freedom (6 DOF) analysis and sEMG data were recorded to understand the effect of wheelchair configurations on the shoulder muscles recruitment and calculated as % Maximum Voluntary Contraction (%MVC). These measurements were taken in the Cardiff University Motion Analysis Laboratory.

The subjects recruited in this study were able-bodied and non-experienced in manual wheelchair propulsion. For transferring the results to the population of persons with a spinal cord injury, one has to keep in mind that due to the potential loss of muscle function the relative muscle activity might as well be higher as reported in this study. Mainly for persons with a high lesion the muscle activity needed for manual wheelchair propulsion is distributed over fewer muscles, which could increase the actual stress on these remaining muscles and could result in higher loading on the shoulder joint.

A limitation of this study was that most of the ten recruited volunteers were of large body mass index. Therefore, as the rear wheels are moved more forward (anteriorly), the distance between the centre of gravity and the rear wheel axle will be shorter and the centre of gravity will get closer to the rear wheel axle. If the rear wheels are too far forward for balance skills of the user then the chair will be at risk of tipping over backwards. This can be considered as a health and safety concern for any person, especially with non-experienced users. Therefore, only posterior displacements were recommended per this study.

Differences in biomechanical results are expected in individuals with disabilities. As compared to the able-bodied and non-experienced subjects, paraplegic and experienced subject presented higher muscle activation during both phases of propulsion. For example, subjects with high level of SCI have less trunk control. In this study, able-bodied participants showed better trunk control as they usually lean their trunk forward during the pushing which generates body momentum to assist the pushing. That may diminish the co-contraction of shoulder muscles during the early stage of recovery.

Although experienced wheelchair users with disabilities have been shown to be more efficient in the wheelchair propulsion task and to differ in the wheelchair propulsion biomechanics that may bring an important understanding to the demands of the task as the typical users, it was more practical to test the used experimental protocol on healthy (non-impaired) subjects first, since they were easier to recruit and call back for repeat measurements when necessary. In addition, a group of controls reduced the variability that would be introduced by a study group with differences in level and completeness of spinal cord injuries.

This study basically explored the question; how upper body joints kinematics and muscle recruitment in manually propulsion users are impacted through adjusting wheelchair axle position? There are a number of differences of the used methodology when compared to other related studies. A marker-based 3D motion analysis technique was used with more recently to the six degrees of freedom (6 DOF) analysis, as an integrated feature in the software that was used to collect the motion capture data (Qualisys Track Manager, QTM, Qualisys, Sweden). This allowed greater flexibility in calculating the joint angles, under a thorough understanding of identifying each segment anatomical coordinate system, in line with the ISB recommendations.

As a novel aspect, this study provided insight into the relationship between wheelchair key configurations like wheel axle positions and the upper limb functionality during manual wheelchair propulsion in terms of quantified difference per cent of the joint kinematics and EMG outcomes between the manufacturer’s set position and adjusted displacements.

Another potential advantage of this study was that the experimental data were collected over ground but not on calibrated wheelchair ergometer or roller dynamometer. Stationary propulsion simulators do not perfectly replicate over ground propulsion. In addition, a standard self-propelled wheelchair was utilised in the study instead of using instrumented or customised devices. All the recruited participants and adjustments were performed on the same device that limited the variability exist per the other related studies. All these aspects tend to provide a flexible acquisition of the manual wheelchair propulsion for able-bodied non-experienced users.

## Conclusion

Movements of the human upper limb are difficult to measure due to the specific structure of this limb. The use of a manual wheelchair for mobility often leads to upper limb pain and associated injury. This study showed an interrelationship between adjusting wheelchair axle position and both upper body kinematic behaviour and muscles activity during manual wheelchair propulsion. It was observed that changing rear wheel axle position posteriorly is correlated with higher upper limb kinematics and muscle activities and so could be linked with higher risks of musculoskeletal disorders.

Appropriate wheelchair axle position should ensure the user’s upper limb joints ROM fall within the normal range. Therefore, when a manual wheelchair user complains of pain due to excessive movement, the clinician could displace the axle position forward to decrease the range of this movement. The reverse situation might apply if the user should experience pain due to an excessive opposite movement. The clinician could try backward displacement of the axle position. Wheelchair configurations that cause joints to use maximum joint ROM considered inappropriate as the excessive movement may cause overuse injury due to joint impingement and tissue undue stress. Therefore, the optimisation of the wheelchair configuration, based on functional characteristics of the user, appears beneficial.

This knowledge may help both manufacturers and clinicians when designing and prescribing wheelchairs that are more proper to users’ functional features, needs and expectations, accordingly profiting users’ independence and quality of life.
